# Critical Reflections on Public Health, Policy and Social Change Toward Healthy Societies: Comment on "How to Build Healthy Societies: A Thematic Analysis of Relevant Conceptual Frameworks"

**DOI:** 10.34172/ijhpm.9135

**Published:** 2025-05-27

**Authors:** Matthew Fisher

**Affiliations:** Stretton Health Equity, Stretton Institute, University of Adelaide, Adelaide, SA, Australia.

**Keywords:** Healthy Societies, Public Well-being, Public Policy, Health Promotion, Public Health Theory

## Abstract

Nambiar and colleagues in this journal identify the main conceptual frameworks offered by public health on *how* to build healthy societies, drawn from key documents published over a span of 50 years. In their analysis they point to strengths and limitations of these frameworks and offer suggestions for their improvement. In this commentary, I argue that both the frameworks on offer and Nambiar and colleagues’ critique are missing important perspectives on well-being itself, on the role of the democratic State, and on the "community arena" and the "policy arena" as two related but distinct arenas for political and social change toward healthy societies.

## Introduction

 Nambiar and colleagues analysed a selected group of key public health reports, evidence reviews and research articles from the last 50 years to identify common concepts of a healthy society and the political means required to achieve it. Following a companion article on conceptual frameworks for the make-up of healthy societies,^1^ this article presents findings on frameworks addressing the question of *how* to build such societies.^2^ The authors identify three “policy levers” directly available to governments and three socio-political “enablers” recommended in the literature as means to drive political and social change toward a healthy society. The levers are regulatory and fiscal measures, intersectoral action, and redefining measures of progress. The enablers are political will and accountability, social mobilisation and community action, and generation and use of knowledge. The authors follow a principle of health equity, recognise social determinants of health (SDH), and base their analysis on the premise that “societal efforts for health are driven by policy levers” (p. 2).

 Nambiar et al give credence to the identified levers and enablers but are also critical of them as largely unchanged over decades (despite limited uptake in policy), largely technical rather than political, lacking evidence in crucial areas, and in need of paradigm-shifting ideas. I have similar concerns about the limitations of these conceptual frameworks, but for different reasons; reasons which resolve into constructive criticism of both the identified levers and enablers, and Nambiar and colleagues’ critique. In short, I argue that both are lacking basic but under-recognised conceptual bases for understanding healthy societies and how to create them. I first outline these conceptual tools drawing on my own recent work on well-being.^3-7^ I then apply these to critique the article and outline alternative ways of thinking about the politics and prosects of healthy societies.

## Conceptualising Problems and Solutions

 The way public health problems are defined is crucial in delimiting preferred “solutions.”^8^ Current public health thinking and research over-relies on epidemiological evidence based on rates of *disease*, defined in biomedical terms.^3^ Thus, while improvements in public health and health equity call for action on SDH, the desired health outcomes typically boil down to changes in absolute or relative rates of disease. It is all too easy for governments to “convert” the problem, framed in these terms, into biomedical, health system “solutions.”^8^ Reducing disease is important, but if we aspire to healthy societies a coherent, shared public health theory of *good health* and *psychological well-being* is sorely needed. I have proposed such a theory, building on convergent evidence across multiple disciplines.^3,6^ In summary, this theory starts with a conception of social intelligence, consisting in brain functions supporting adaptive behaviour is social settings. In this model, acute stress arousal assists flexible behaviour change in response to social cues. However chronic stress and risks to mental health ensue when environmental stressors are present recurrently, and the subject cannot see a way to resolve or avoid them. Well-being is then defined in two parts, as the ability: to self-regulate social intelligence and behaviour to cope with minor stressors, avoid chronic stress, and engage in positive social relationships; and to balance purposeful goal-directed action with other states of being, which reduce stress and enable self-awareness, calm, happiness, connection, and meaning. Well-being as “ability” is realised (or not) through the interactions between personal and social resources and social-environmental settings.^3,6^

 This theoretical work presents the political problems and solutions of a “healthy society” in different terms to those in Nambiar and colleagues’ article, resists a biomedical interpretation, and appeals to a growing political and social interest in well-being. However, the response from the discipline of public health has largely been silence.

## Political Paradigms

 As Nambiar et al acknowledge, the political ideology and prevailing paradigm driving crises in socioeconomic inequality, psychosocial distress, and other areas is neoliberalism.^6^ The central philosophical and ideological fulcrum defining the difference between genuine social democratic governance (needed for healthy societies) and neoliberalism concerns the *role of the State* (ie, the enduring institutions of government).^6^ Thus, I would argue, an essential paradigm shift in political thinking needed to counter neoliberalism and promote healthy societies is to revive the foremost ethical duty of the democratic State to *serve the public interest,*^6,9^ in which public well-being and ecological sustainability *must* figure as enduring first-order priorities. If the public interest conflicts with private interests in a market economy, the State’s duty is the put the public interest first.

## Arenas of Change

 Longstanding concepts in public health literature define two related but distinct *arenas* for action on public health. This distinction is highly apposite to understanding the ‘how’ of change toward a healthy society but is never made explicit in Nambiar and colleagues’ article. Founding documents such as the *Declaration of Alma Ata*^10^ and the *Ottawa Charter for Health Promotion*^11^ describe the *actual* social production of health and well-being in processes of human development and mutuality occurring in localised spaces, albeit that these are supported and enabled by governments (Let us call this “the community arena”). Similarly, conceptual frameworks on SDH recognise that the *actual* impacts of determinants occur in the proximal (localised) circumstances of people’s lives,^12^ while political determinants distribute access or exposure to healthy or unhealthy circumstances.^13^

 When conceptualising the “how” of healthy societies, emphasis on health equity or the policy arena can overstate the role of governments (including policy for distribution), while downplaying the equally important matter of the actual conditions required for health and well-being, including Alma Ata’s emphasis on community-based primary healthcare,^10^ and Ottawa’s emphasis on supportive environments, empowered communities, and personal skills.^11^ To an extent, Nambiar and colleagues’ article reproduces this problem. I have recently published work applying the public health theory of well-being outlined above to reiterate and expand on the essential localised conditions for psychological well-being.^4-6^ Of course, governments have a crucial role to ensure universal access to these conditions.^6^

 The paradigm shift required here is to recognise that the *foundations* of a healthy society lie in the localised spaces—the “community arena”—where health and well-being are produced, and that the essential role of governments and public policy can be better defined when understood in this context.^6^

## Policy Levers

 As Nambiar and colleagues’ analysis displays, the fiscal and regulatory measures proposed in public health literature tend to focus on reducing socioeconomic inequalities and exposures to unhealthy corporate products and practices. These proposals are made for sound public health reasons but are not enough. Somewhat improved access to resources or healthier products set within the continuing context of a neoliberal society do not add up to a healthy society. An anchoring conception of health and well-being in the community arena offers a more defined conception of the *systemic changes *to which fiscal and regulatory measures ought to contribute.

 The abiding problems with conceptions of intersectoral action for public health are similar. The *integrative* conception of healthy social conditions does not lie merely in cooperation between government agencies as such, no matter how well intended. It lies in the integrated, multi-faceted design and conduct of healthy communities, tailored to different places.^5,14^ Centring one’s conception of a healthy society on the community arena in this way provides much needed definition and direction to the varying *roles* of public agencies and strongly indicates a need for more localised governance structures.^5,15^ Without such direction, I see no sign of intersectoral action between public agencies overcoming institutional interests, much less spontaneously resolving into a coherent set of contributions to a healthy society. To expect it would, is putting the cart before the horse.

 On the question of redefining measures of progress, the tacit supposition that governments will always act on what they measure is just not borne out in practice. Even if such measures are enacted in policy, the default method is likely to be to assign them as performance targets across a range of still-siloed government agencies, reiterating the weaknesses of “intersectoral action” in another form. Either measures are attached to an effective method, or they are not.

## Policy Enablers

 Lack of political will and accountability, and power asymmetries are of course difficult, related problems. The key to change lies with well-informed social movements motivated by a shared vision for a healthy society and growing public awareness of political failures. The role of public health is to participate in such movements while also trying to hold governments accountable. I believe concepts of individual and community well-being, and of the public interest role of the State have a valuable role to play in these endeavours.

 Nambiar et al seem to see social mobilisation and community action only in terms of generating public demands for changes in policy. They overlook the potential for parallel community actions to defend, reclaim and remake supportive conditions for well-being in localised spaces.^4,15^ Social movements for a healthy society cannot be sustained only on anger with what is; they also need real and practicable ideas about what can be.

 On the generation and use of knowledge, yes, research has a role of play. However, the authors’ analysis may reflect uncritical academic assumptions about the value of “more research.” I am sceptical that more research on power, policy-making, “what works” (p. 7) or SDH “in specific, actionable contexts” (p. 5) will add much to what we already know or dramatically shift policy thinking. My theoretical work on well-being—for its part—does not call for “more research” but argues the value of drawing together existing evidence to build theory with explanatory power across multiple policy issues.^6^

## The Discussion

 In their discussion Nambiar et al criticise the literature analysed for insufficient considerations of politics and power, and lack of evaluation of policies to assess what has “worked or not worked” (p. 7) in a manner relevant to healthy societies. Neither of these are unimportant issues to consider in the challenge of charting a public health contribution to healthy societies. However, as the paragraph above attests, I am sceptical. The assumption that more and more ostensibly “new” knowledge—squeezed into tiny “gaps” in the literature—is useful by definition, has run its course. Public health researchers must critically assess their own contributions and concepts in overtly political terms. We understand the essence of “what works” for a healthy society when we understand well-being and the conditions required to create it, and we can do that with the knowledge we already have.^6^

 To enact well-being policy in the community arena, public health and policy leaders should adopt a place-based approach, collaborating with community actors to deliver strategies in key areas such as: comprehensive primary healthcare; early child development; education for lifelong learning; meaningful work; social connectedness; care for nature; healthy food; secure housing; healthy neighbourhood design; and creative practices.^4,5^

 On the need for paradigm shifts, positive visions, and clearer articulation of “what a good life could be … [and] how this could be achieved” (p. 7), I can only strongly agree.^6^

## Ethical issues

 Not applicable.

## Conflicts of interest

 Author declares that he has no conflicts of interest.

## References

[1] Buse K, Bestman A, Srivastava S, Marten R, Yangchen S, Nambiar D (2023). What are healthy societies? A thematic analysis of relevant conceptual frameworks. Int J Health Policy Manag.

[2] Nambiar D, Bestman A, Srivastava S, Marten R, Yangchen S, Buse K (2023). How to Build Healthy Societies: A Thematic Analysis of Relevant Conceptual Frameworks. Int J Health Policy Manag.

[3] Fisher M (2019). A theory of public wellbeing. BMC Publ Health.

[4] Fisher M (2021). Moving social policy from mental illness to public wellbeing. J Soc Policy.

[5] Fisher M (2023). Multi-sectoral action to promote psychological wellbeing: Theorising the role of place-based policy. Health Promot J Austr.

[6] Fisher M. How to Create Societies for Human Wellbeing: Through Public Policy and Social Change. Bristol, UK: Policy Press; 2024.

[7] Fisher M, Baum F (2010). The social determinants of mental health: Implications for research and health promotion. Aust NZ J Psychiatry.

[8] Fisher M, Baum F, MacDougall C, Newman L, McDermott D (2016). To what extent do Australian health policy documents address social determinants of health and health equity?. J Soc Policy.

[9] Wheeler C. The Public Interest: We Know It’s Important, But Do We Know What It Means. Canberra: Australian Institute of Administrative Law; 2006.

[10] World Health Organization. Declaration of Alma-Ata. Alma-Ata. USSR: International Conference on Primary Health Care, World Health Organization; 1978.

[11] World Health Organization. First International Conference on Health Promotion - Ottawa Charter for Health Promotion (editor); November 21, 1986; Ottowa, Canada.

[12] Singh-Manoux A, Marmot M (2005). Role of socialization in explaining social inequalities in health. Soc Sci Med.

[13] Solar O, Irwin A. A Conceptual Framework for Action on the Social Determinants of Health. Geneva: World Health Organization; 2010.

[14] Giles-Corti B, Vernez-Moudon A, Reis R (2016). City planning and population health: A global challenge. Lancet.

[15] UCL Institute of Health Equity. What Are Marmot Places? London: University College London; 2024.

